# Global compilation of bioavailable strontium isotope data

**DOI:** 10.1038/s41597-026-06643-3

**Published:** 2026-01-24

**Authors:** Chris Stantis, Malte Willmes, Mael Le Corre, Hannah F. James, Camilla Kafino, Kirsten A. Verostick, Elizabeth H. Paris, Justin Bank, Gabriel J. Bowen, Kévin Salesse, Clément P. Bataille

**Affiliations:** 1 School of Anthropology, Political Science, and Sociology, Southern Illinois University, Carbondale, Illinois USA; 2 Department of Geology and Geophysics, University of Utah, Salt Lake City, Utah USA; 3 Norwegian Institute for Nature Research, PO Box 5685, 7485 Torgarden, Trondheim Norway; 4 UMR 7209 - Archéozoologie et Archéobotanique-Sociétés, Pratiques et Environnements, Muséum National d’Histoire Naturelle, Paris, France; 5 Archaeology, Environmental Changes & Geo-Chemistry Research Group, Vrije Universiteit Brussel, Ixelles, Belgium; 6 National Institute of Criminalistics (INC), Federal Police of Brazil, Brasília, Brazil; 7 Department of Anthropology and Archaeology, University of Calgary. 2500 University Drive NW, ES 356, Calgary, AB T2N 1N4 Canada; 8 School Of Earth Systems And Sustainability, Southern Illinois University, Carbondale, Illinois USA; 9 Department of Anthropology, Faculty of Science, Masaryk University, Brno, Czech Republic; 10 Department of Forestry and Natural Resources, Purdue University, Lafayette, USA; 11 Department of Earth and Environmental Science, University of Ottawa, Ottawa, Canada

**Keywords:** Biogeochemistry, Ecology, Anthropology

## Abstract

Bioavailable strontium isotope ratios (^87^Sr/^86^Sr) have broad applications for geolocation and movement reconstructions in ecology, paleontology, archaeology, forensics, and environmental and food sciences. These applications require a comprehensive ^87^Sr/^86^Sr reference dataset to identify isotopically distinct areas. Bioavailable ^87^Sr/^86^Sr reference datasets have been created for many regions world-wide and are based on a large range of different sample materials including soil, water, flora, and fauna. Here we compiled and harmonized 28,347 published bioavailable ^87^Sr/^86^Sr data from over 150 countries representing decades of work by hundreds of researchers to provide a global dataset as a basis for reference and isoscape development. These data provide researchers across many different disciplines with a common reference dataset to use as a foundation for their research projects. Metadata and method details are provided to allow for the individual assessment of the data. The dataset is available at two community data repositories dedicated to stable isotope data, IsoArcH and IsoBank.

## Background & Summary

Strontium isotope ratios (^87^Sr/^86^Sr) are used in many fields for source attribution and geolocation applications^1^. These applications are reliant on the principle that these ratios record the movement of matter within environmental systems, and thus the isotope ratios of these substances can be compared with reference data or spatial models to estimate the geographic origin of the material^2^. For non- or slowly-recycling living/once-living tissues (e.g., hair, feather, teeth, bones), their ^87^Sr/^86^Sr can be reflective of the physical environment when these tissues were forming^3^.

^87^Sr/^86^Sr tracing has been widely used and holds enormous potential across disciplines; it has been used in environmental sciences from identifying the geographic origin of soil nutrients^4,5^ to characterizing weathering in river systems^6^, in ecology from tracing insect migration to reconstructing extinct megafauna mobility^7,8^, in food and forensic sciences from certifying wine origin^9^ to tracing illegal drugs^10,11^, or in anthropology from identifying the remains of unidentified decedents^12^ to exploring migration and trade networks in past populations^13^. Ecology, paleontology, archaeology, and forensic studies have all used the principle that living tissues will represent the bioavailable ^87^Sr/^86^Sr of their local geology, or at least the underlying geology from which their food was grown^14–16^. One key limiter to ^87^Sr/^86^Sr data is that it can never be interpreted in a vacuum– reference data is key to verifying and quantifying local ^87^Sr/^86^Sr (or, as is more typically the case in geolocation using isotopes, exclusion of potential localities).

Large-scale open databases allow the re-use of data to address questions beyond the scope of the original research that generated the data. Beyond utility, ethical implications are present in isotope data’s curation and future use. For any scientific data, sharing research data on open science platforms reduces the gap between those researchers that are able to afford data collection/storage and those who are not^17–19^. Moreover, stable isotope analysis predicates the destruction of some part of a sample. This may be less ethically weighted in some fields (e.g., hydrology, geoscience), but rare and irreplaceable samples such as archaeologically-derived human remains necessitate extra care in mitigating the impact of isotopic analyses’ destructive nature via the reuse of collected data when possible^20,21^. Data sharing maximizes the impact and potential benefit of any analyses conducted on such samples.

Recognition of the importance of isotopic data-sharing is reflected in the recent emergence of two large community-driven platforms for isotope data archival, IsoArcH and IsoBank. IsoArcH was created initially to hold stable isotope data from Mediterranean archaeological projects^22^, but grew to a global scale as other archaeological scientists around the world began helping in community-led ingest of previously published data^23^. IsoBank envisions a cross-disciplinary user platform for any research that uses stable isotope data^24–26^. Both IsoBank and IsoArcH are responses to the increased output in publications containing vast amounts of isotopic data in the last few decades and both offer some guidelines on how to share isotopic data in interoperable ways.

The goal of collating ^87^Sr/^86^Sr data from floral, faunal, water, and geological samples arose from recognition of the opportunity provided by these repositories to compile, harmonize, and make discoverable the large and diverse volume of bioavailable ^87^Sr/^86^Srdata in the literature. A secondary goal presented itself from the question of how the two repositories handled ingestion/integration of this type of isotope data as well as future extraction for third-party re-use. To this end, we the authors, as stable isotope scientists with affiliations to both repositories, joined in Project ARDUOUS (Aligning Repositories/Databases by Utilizing Optimized Upload Strategies) to create this data product.

The main expected output using ARDUOUS data will be the development of baseline models predicting bioavailable ^87^Sr/^86^Sr in regions of interest. An associated R package is being developed by the ARDUOUS team to provide the code necessary to use random-forest modeling to generate a strontium isoscape using this data. As part of living repositories, users can also contribute more data to IsoArcH and IsoBank, enriching the potential usage noted here.

**Fig. 1:**
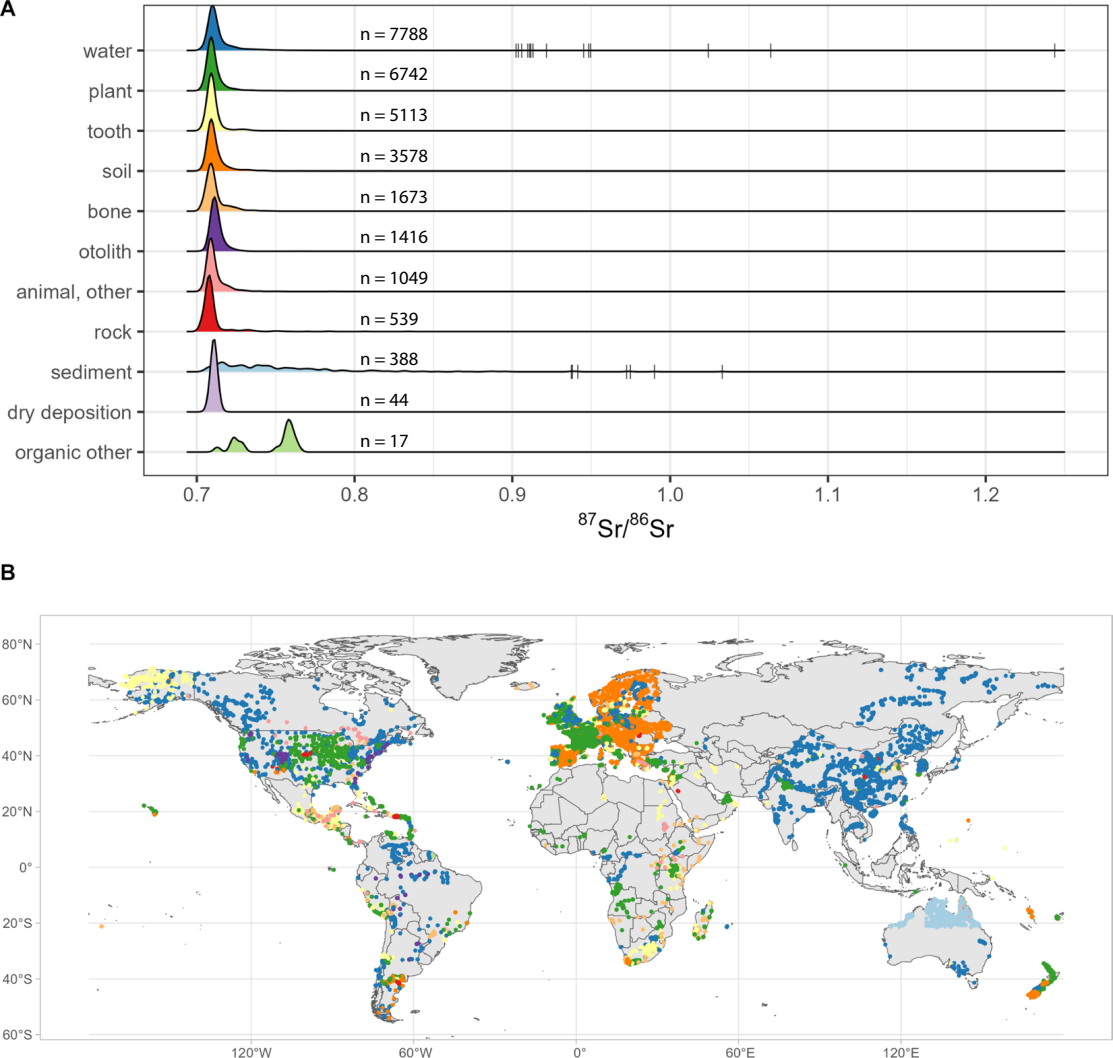
(**A**) Density plots of ^87^Sr/^86^Sr values for the data product, by generalized sample type. (**B**) Global map of geolocated points within Project ARDUOUS, by simplified sample type.

Notable spatial gaps exist across the globe (Fig. 1). For example, while Europe is well characterized (largely due to an interest in origin verification of food products), there are notable gaps in large swathes of Australia, northern Africa, and western Asia. Future research could be targeted at these gaps, with the current collated data product used as evidence of systematic review of available data.

## Methods

### Literature review

The data collated for this project come from previously published research, including peer-reviewed works, theses and dissertations, and reports and other grey literature. A systematic literature review across all disciplines that generate bioavailable ^87^Sr/^86^Sr data was conducted. We searched Web of Science and Google Scholar using the keywords “strontium isotopes”, “^87^Sr/^86^Sr”. We also used previously published collations of data to identify peer-reviewed publications and other data sources^15,27–29^, especially those covering outputs written in languages in which our team members were not fluent. The initial dataset began with a 2020 global compilation^15^, and this study then focused on systematically finding any studies published since then or missed by the original review. Research producing ^87^Sr/^86^Sr from non-bioavailable sources (e.g., petrological and mining studies) were not included in the project bibliography. Bioavailable ⁸⁷Sr/⁸⁶Sr data derived from surface rock samples were not comprehensively compiled; instead, they were included only when reported in studies that simultaneously provided bioavailable ⁸⁷Sr/⁸⁶Sr data from other substrates.

After deduplication, 474 bibliographic references were gathered for data extraction^5,6,11,15,27–499^. To aid in reproducibility and correct attributions of those original data generators, all data provenance are documented as: (1) citation references and (2) DOIs where available. Thus, all data is traceable to the original published source, and we request the citation of these original sources when the data are reused.

### Data ingestion

The data were harmonized for ingestion. The data definitions for Project ARDUOUS were largely derived from the metadata guide created by IsoBank^500^; some variables needed to be created that were specific to the needs of ^87^Sr/^86^Sr analysis, especially within the contexts of creating bioavailable “isoscapes”– continuous spatial models of isotopic variation. All data definitions are available in the metadata guide, but we explain the additional variables needed for this project.

#### Reported reference value

The main internationally certified reference material used in laboratories to validate analytical methods is Standard Reference Material (SRM) 987, produced by the National Institute of Standards & Technology^501^. Sometimes known as NIST 987 or NBS 987, this reference material has been reported as having a different measurement from the original certified absolute abundance ratio. In publications, analysts report the measured and standard deviation ratios of SRM 987 samples analyzed during the analytical session and compare it to the certified reference ratio. Generally the difference between analyzed and certified ratios are small. Depending on laboratory practices, measured ratios are either left as is or corrected so that measured SRM 987 ratios fit the certified ratio. To ensure that all data can be replaced on the same *Reported Reference Value* for NIST 987, we compile the measured ^87^Sr/^86^Sr ratios of SRM 987 when reported.

#### Sr Concentration and Sr Concentration Error

In addition to isotopic composition, Sr concentration reported in ppm can be used as a tracer in hydrological, geological, and ecological studies^502^.

#### Georeferencing method

To create strontium isoscapes, all data needs to be georeferenced with latitude and longitude coordinates. Three georeferencing methods were categorized for this project: GPS, Map Georeferencing, and Site Name Georeferencing.

Publications could provide geolocation data as part of the tabulated report, requiring, in some instance, a conversion of their coordinate systems into decimal degrees that we used in our data product. These coordinates were labeled ‘GPS’. In other publications, authors reported their data in the form of a map with points. These maps had to be interpreted by project ARDUOUS team members; PlotDigitizer^503^, an online tool for extracting data from uploaded graphs, could help with estimating coordinates. These were categorized as “Map Georeferencing”. Finally, in some other publications, especially in the case of archaeological and paleontological studies which focused on a few excavated sites, only site names were provided; these sites could generally be found online and coordinates estimated. In these instances, the Georeferencing Method was “Site Name Georeferencing”.

Some reported data were converted for standardization in our data product. Common conversions include:

- Converting 2 SD error to 1 SD error for the standard deviation of the reference material and quality control material in an analytical run
- Converting reported Sr concentrations to ppm. Other common concentration units of Sr were ppb, μmol/L and mg/L
- Harmonizing date values to MM/DD/YYYY as per IsoBank guidelines
- Conversion of coordinates to decimal degrees

## Data Record

Project ARDUOUS data are integrated within both IsoArcH and IsoBank repositories. At either repository, the data created for this project can either be downloaded as a single dataset, or found and downloaded in part with other datasets using filter functions designed for the repository (e.g., download only a certain material type such as soil). Both repositories contain detailed data dictionaries for all variables included in this dataset.

## Technical Validation

### Input validation

Checks to validate the data collection and conversion procedures were utilized to help reduce the occurrence of human error during data ingest. Most data were cleaned, examined, and transformed using OpenRefine, an open-source software^504^. Data collated by each co-author was first checked column by column to ensure that required variables were provided, and that, where applicable, values aligned with controlled terms. With most co-authors focusing on a single broad region (e.g., finding all data reporting Australian bioavailable strontium), mapping the latitude/longitude ensured there were no errors regarding ratios that could not possibly match the region. Naturally-occurring ^87^Sr/^86^Sr on Earth range from ~0.700 to 1.300, and so ratios outside this range were identified as potential input errors (e.g., ratios created from keystroke errors such as ‘7.092’ instead of ‘0.7092’). Generally, data are input exactly as published. The only exception is from a dataset created by Killgrove and Montgomery^57^; an error in that paper was communicated to the Project ARDUOUS team during upload and was therefore corrected.

### Analytical validation

All available published ^87^Sr/^86^Sr data were integrated into the final data product. Primary reference material and reported reference material ratios are provided for each datapoint when known. Key laboratory preparation steps that might affect expected ratio outcomes are noted in the variable *Preparation Step* (e.g., digestion, dissolution, acidification).

## Usage Notes

At IsoArcH, the link provided in this article leads to a webpage with clear locations for downloading the dataset and the accompanying.ris for the references list. At the IsoBank repository, all data are stored at the link provided in this article. To download the data as a .csv file in IsoBank’s current form, we recommend the user navigate to the ‘Dataset List’ webpage, find the ARDUOUS submission, and click the hyperlink ending in ‘.csv’.

With the variable *Scientific Name*, animals that are known to have large migration patterns and thus might reflect wide geographic areas can be filtered. All humans and other *Homo* genera, whether relatively modern or from archaeological/paleoanthropological contexts, might also not be reflective of their burial place but are included in this data product. This is also an important consideration for a small proportion of modern and archaeological faunal remains, as both live animals and animal products can also be exchanged over long distances, resulting in strontium ratios that are similarly not reflective of their recovery location.

Regardless of ultimate research applications, we encourage end-users to cite the original published data sources that are used rather than solely this project. The data compiled by project ARDUOUS represent the efforts of researchers collecting samples in 153 countries documented in 474 studies. These data were collected for a broad range of initial purposes, and the full context of sample collection and treatment cannot be documented in a global compilation of the type presented here. For this reason, and to ensure traceability and recognition of the contributions of the original authors, we request that users of the ARDUOUS data compilation review and cite the original sources for data that they reuse in their own projects. To facilitate this, ARDUOUS provides a single .RIS file incorporating all the references from the compiled database, available via IsoArcH^505^.

## Data Availability

The data are openly available at two data repositories. They are available in IsoArcH at 10.48530/isoarch.2024.002, as well as in IsoBank https://isobank.tacc.utexas.edu/analyses/submitted_dataset_analyses_list/932.

## Code availability

The code used to summarize data and generate the map and figures are available via GitHub (10.5281/zenodo.15786264)^506^.
